# The hTH-GFP Reporter Rat Model for the Study of Parkinson's Disease

**DOI:** 10.1371/journal.pone.0113151

**Published:** 2014-12-02

**Authors:** Lorraine Iacovitti, Xiaotao Wei, Jingli Cai, Eric W. Kostuk, Ruihe Lin, Alexander Gorodinsky, Philip Roman, Gretchen Kusek, Sonal S. Das, Audrey Dufour, Terina N. Martinez, Kuldip D. Dave

**Affiliations:** 1 Farber Institute of Neurosciences, Department of Neuroscience, Thomas Jefferson University, Philadelphia, Pennsylvania, United States of America; 2 Taconic Farms, Inc., Hudson, New York, United States of America; 3 The Michael J. Fox Foundation for Parkinson's Research, New York, New York, United States of America; Prince Henry's Institute, Australia

## Abstract

Parkinson disease (PD) is the second leading neurodegenerative disease in the US. As there is no known cause or cure for PD, researchers continue to investigate disease mechanisms and potential new therapies in cell culture and in animal models of PD. In PD, one of the most profoundly affected neuronal populations is the tyrosine hydroxylase (TH)-expressing dopaminergic (DA) neurons of the substantia nigra pars compacta (SNpc). These DA-producing neurons undergo degeneration while neighboring DA-producing cells of the ventral tegmental area (VTA) are largely spared. To aid in these studies, The Michael J. Fox Foundation (MJFF) partnered with Thomas Jefferson University and Taconic Inc. to generate new transgenic rat lines carrying the human TH gene promoter driving EGFP using a 11 kb construct used previously to create a hTH-GFP mouse reporter line. Of the five rat founder lines that were generated, three exhibited high level specific GFP fluorescence in DA brain structures (ie. SN, VTA, striatum, olfactory bulb, hypothalamus). As with the hTH-GFP mouse, none of the rat lines exhibit reporter expression in adrenergic structures like the adrenal gland. Line 12141, with its high levels of GFP in adult DA brain structures and minimal ectopic GFP expression in non-DA structures, was characterized in detail. We show here that this line allows for anatomical visualization and microdissection of the rat midbrain into SNpc and/or VTA, enabling detailed analysis of midbrain DA neurons and axonal projections after toxin treatment in vivo. Moreover, we further show that embryonic SNpc and/or VTA neurons, enriched by microdissection or FACS, can be used in culture or transplant studies of PD. Thus, the hTH-GFP reporter rat should be a valuable tool for Parkinson's disease research.

## Introduction

Currently, there are a number of transgenic mouse lines that are used to study Parkinson's disease (PD), including those in which rodent [1]–[3] or human [4], [5] tyrosine hydroxylase (TH), dopamine transporter (DAT) [6] or DA transcription factor (Pitx-3) [7] promoters have been engineered to drive expression of EGFP reporter protein expression in midbrain dopamine (DA) neurons of the substantia nigra pars compacta (SNpc) and ventral tegmental area (VTA) and in their respective terminals in the striatum and cortex. These mice have allowed researchers to study Pitx-3+ DA neural progenitors [7], immature TH+ and mature DAT+ DA neurons [6]–[11] in vitro. In vivo, these models have enabled the study of PD, particularly when systemic MPTP is used to generate damage to DA neurons.

However, the most well-established and commonly used animal model of PD remains the 6-hydroxydopamine (6-OHDA) lesioned rat, first described by Ungerstedt [8]. Because of the larger size of the rat brain as compared to the mouse brain, this model allows local administration of toxin unilaterally into the SNpc, striatum or median forebrain bundle (MFB), resulting in ipsilateral motor deficits which can be assessed, quantified, and compared to the contralateral side over time and following various experimental treatments. Because of the ease and reliability of this behavioral model [9], [10], it has been long-favored by researchers. However, until now, there has been no transgenic reporter rat line to facilitate these studies in vivo or in vitro. The Michael J. Fox Foundation (MJFF) has developed a strategy to directly sponsor the generation, phenotypic characterization and distribution of preclinical rodent models in order to aid and accelerate PD research [11]. As part of this overall strategy, MJFF, partnered with Thomas Jefferson University and Taconic Inc. to generate new transgenic rat lines carrying 11 kb of the human TH gene promoter driving EGFP [5]. With its high levels of GFP, hTH-GFP rat reporter line 12141 allows for anatomical visualization of the rat SNpc and/or VTA and detailed analysis of midbrain DA neurons and axonal projections after toxin treatment in vivo. Moreover, embryonic microdissection of fluorescent SNpc and/or VTA greatly enriches the proportion of DA neurons that can be isolated from each of these midbrain regions for culture and transplant studies. With further FACS analysis, DA neurons from each of these regions can be purified to near homogeneity. Thus, the hTH-GFP reporter rat should be a valuable tool for Parkinson disease research.

## Methods

### Animals and IACUC Policies

All animals used in this study were maintained in accord with the Office of Animal Resources at Thomas Jefferson University. The protocols were approved by the Institutional Animal Care & Use Committee (IACUC) at Thomas Jefferson University, protocol # 457A, 457K. Surgery rats were anesthetized with Ketamine (80 mg/ml)/xylazine (5 mg/ml)/acepromazine (1.6 mg/ml). Rats were sacrificed with Nembutol(40 mg/kg) followed by perfusion with 4% Paraformaldehyde.

### Generation of Transgenic Rats

Plasmid phTH-11 kb-EGFP (pMAK 288–12) places the hTH promoter upstream of the EGFP (GFP) reporter gene [5]. DNA fragments including 10.794 kb of the distal hTH promoter, 1.168 kb of proximal hTH promoter were ligated to produce plasmid phTH-11 which was placed upstream of the GFP reporter gene.

The 12,007 bp NotI-AflII restriction fragment from plasmid phTH-11 kb-EGFP was isolated by electroelution, precipitated from ethanol/1 M ammonium acetate, washed with 75% ethanol, dried, and resuspended in TE buffer. This DNA was microinjected into single cell embryos of SD (Sprague Dawley) rats. Transgenic pups were identified by PCR, which detects the presence of the eGFP open reading frame. To amplify a 264 bp product the following primers were used: CAGCACGACTTCTTCAAGTCC and GATCTTGAAGTTCACCTTGATGC.

### Transgene Copy Number Analysis

DNA copy numbers of the inserted 12,007 bp NotI-AflII restriction fragment were measured by real-time qPCR with the primers ggacggcgacgtaaacg and gtgcagatgaacttcagggtca and the probe 6FAM-cgtgtccggcgagggcga-BBQ (TIB MOLBIOL Syntheselabor GmbH, Berlin, Germany) specific for eGFP. As a reference, the Tert specific primers tgcgggtcacccctgtac and ggacccaaggttagtgaaattcc and the probe YAK-ttgtgccaccacgatacctggt-BBQ were used. DNA from ear punch samples of the transgenic rat lines was isolated and quadruplicates of 10 ng were analyzed using the depicted assays and the TaqMan Gene Expression Master Mix on a TaqMan HT7900 (Applied Biosystems, Foster City, CA). Copy numbers were calculated using the Copy Caller Software v2.0 (Applied Biosystems, Foster City, CA).

### Immunocytochemistry/Cell Counts

Adult rat brains were perfused with cold 4% paraformaldehyde. Embryonic brains (E10.5-E18.5) from TH-GFP rats were immersion fixed in 4% paraformaldehyde overnight. Brains were washed with 1xPBS and transferred to 30% sucrose solution in 1xPBS and mounted in O.C.T. for sectioning. Adult brains were sectioned at 30 µm on a Leica freezing microtome and embryonic brains of various stages were sectioned at 15 µm on a Microm cryostat as described in [12]. Sections were processed with primary antibodies (rabbit-anti-TH 1∶200, Pel Freeze, chicken-anti-GFP 1∶200, Millipore) at 4°C overnight using procedures for immunocytochemistry as described previously [13]. All secondary antibodies were AlexaFluor antibodies from Invitrogen used at 1∶200 for 30 min at room temperature. Slides were covered with ProLong Gold antifade reagent (Invitrogen). Cultures were rinsed twice and then fixed with 4% paraformaldehyde and stained with primary antibodies at 4°C overnight as previously detailed [14]. Cultures were also counter stained with Dapi (Molecular Probes) at 1∶1000. Stained cells and sections were examined using an OlympusIX81 Image Analysis System.

### Tissue Culture

Timed pregnancies were performed using either wild type or TH-GFP^+^ female rats mated with TH-GFP^+^ males. Embryonic day zero (E0) was determined as the first day a vaginal plug was visible. On E14.5, embryos were harvested and placed in ice cold DPBS (Gibco #14190-144; no CaCl_2_ or MgCl_2_). GFP^+^ pups were visualized under a dissection microscope (Nikon SMZ1500; adjustable 1-11.5x objective) by using a high pressure mercury lamp with a 495 nm filter attachment. The midbrain was surgically extracted, cleaned of non-GFP^+^ tissues and the VTA and SN were carefully separated. Tissues were collected and placed in 1.5 mL warmed Accutase (Sigma-Aldrich, cat # A6964) for up to 30 minutes. Solution was gently agitated every 5 minutes to aid in dissociation. NEP Basal media (DMEM/F12, B-27 & N2 Supplements (Gibco), 1 mg/mL BSA, and Penn/Strep) was added to terminate the enzymatic reaction. Tissues were then briefly spun at 1000 rpm for 2 minutes and the supernatant media was aspirated. Tissues were re-suspended in culture media (NEP Basal media +5% FBS) with 10 M Rock Inhibitor (Y-27632, Calbiochem #688000) for 10 minutes prior to mechanical dissociation. For mechanical dissociation, a 1 mL pippetor was used with low retention tips and tissues were gently triturated 8–10 times. Cells were then pelleted for 5 minutes at 1000 rpm, re-suspended in culture media and counted on a hemocytometer. Cells were plated in a microdrop on 8-well chamber slides pre-coated with poly-D-lysine (1 mg/mL, overnight at 4°C) at a density of 2.5–3.5×10^5^ cells in approximately 30 L of culture media. Cells were allowed to attach to the substrate before media was added to final volume of 200 L per well.

### FACS Sorting Protocol

All preparation and transport procedures were carried out on ice. Dissociated cells, generated as described above were pelleted and re-suspended in 1 mL DPBS with 5% FBS. Cell suspensions were passed through a 100 µm cell strainer to eliminate any large clumps or debris. A small aliquot (approximately 30 µL) of both control (GFP^-^) and GFP^+^ cell suspension were plated down before sorting. A GFP^+^ calibration control was prepared for the FACS sorter by removal for 100 µL of GFP^+^ cell suspension added to 900 µL DPBS+5% FBS. The same procedure was used for GFP^-^ cells to create a GFP^-^ control sample for calibration. To the remainder of the GFP^+^ sample, 100 uL DPBS+5%FBS and 1 uL Propidium Iodide (PI) was added as an index of cell viability and separation of PI^+^/GFP^+^ (dead) and PI^-^/GFP^+^ (live) cells. Cells were sorted on a BD Biosciences FACSAria and collected in two tubes with 2 mL of culture media. After sorting, cells were spun down for 5 minutes at 1000 rpm. The cell pellet was re-suspended in culture media and counted with a hemocytometer prior to plating. Sorted cells (2.5–3.5×10^5^ cells/30 µL) were plated onto Geltrex pre-coated 8-well chamber slides as above. After growing 3 days in 5% FBS-containing media, cells were fixed and immunostained for TH.

### MPP+ Studies

To create PD-like damage, cultures of SNpc or VTA derived cells were plated at 300,000cells/well and grown for 7 days in defined media. Cultures were then treated with PBS or 10 µM and 50 µM MPP+ (Sigma: Cat# D048) for 48 hours. Control and MPP+-treated cultures were then fixed, stained for TH and DA neurons counted in 5 representative fields in triplicate cultures and expressed as the average ± SEM.

### 6-OHDA Lesions

Animals and IACUC policies. As described previously [13], 5 male and 5 female hTH-GFP rats were maintained in accord with the Office of Animal Resources at Thomas Jefferson University were made parkinsonian for these studies. Briefly, rats were anesthetized with Ketamine (80 mg/ml)/xylazine (5 mg/ml)/acepromazine (1.6 mg/ml), placed in a stereotaxic apparatus (Kopf Instruments) and a 26-gauge Hamilton syringe containing 6-OHDA (Sigma; 20 µg/ml in 4 µl PBS containing 0.2 mg/ml ascorbate) was lowered into the right median forebrain bundle (AP: -4.4 mm, ML: −1.2 mm, DV: −7.8 mm from bregma). The 6-OHDA solution was gradually injected at a rate of 1 µl/min. All lesions were verified 3 and 6 weeks later by assessment of rotational behavior in an automated rotometer system (Columbus Instruments) following amphetamine challenge (5 mg/kg, i.p.).

### Transplantation

Animals with verifiable 6-OHDA lesions (>10 ipsilateral turns/min) were implanted with E14.5 hTH-GFP cells microdissected from the GFP+ ventral midbrain area. Cells were implanted into the striatum on the side ipsilateral to the lesion using procedures described previously [15], [16]. A total of 5×10^5^cells in10 µl of DMEM +0.05% of DNaseI was stereotaxically injected, depositing a third of the cells at three depths (ML: −2.7 mm, DV: −5.2, −4.7, −4.2 mm). Animals were then sacrificed at 4 days (n = 4) or 4 weeks (n = 4) following transplantation. In the 4 week group, motor behavior was re-assessed by rotation test at three weeks after transplantation. Quantification of surviving TH+ neurons within grafts was performed on every eighth section where a graft was identifiable. Cell counts were analyzed by Image J.

### Statistical Analysis

DA neuron loss after MPP+ treatment in vitro and behavioral differences before and after transplantation of GFP+ neurons in vivo were analyzed. Results were presented as the mean ± SEM. The statistical significance of the mean difference was calculated using the two-sample Student's t-test in vitro and paired samples t-test in vivo. A p-value <0.05 was considered significant.

## Results

### Detailed Analysis of TH and GFP in Line 12141 and Comparison with Other Founder Lines

**Figure 1:**
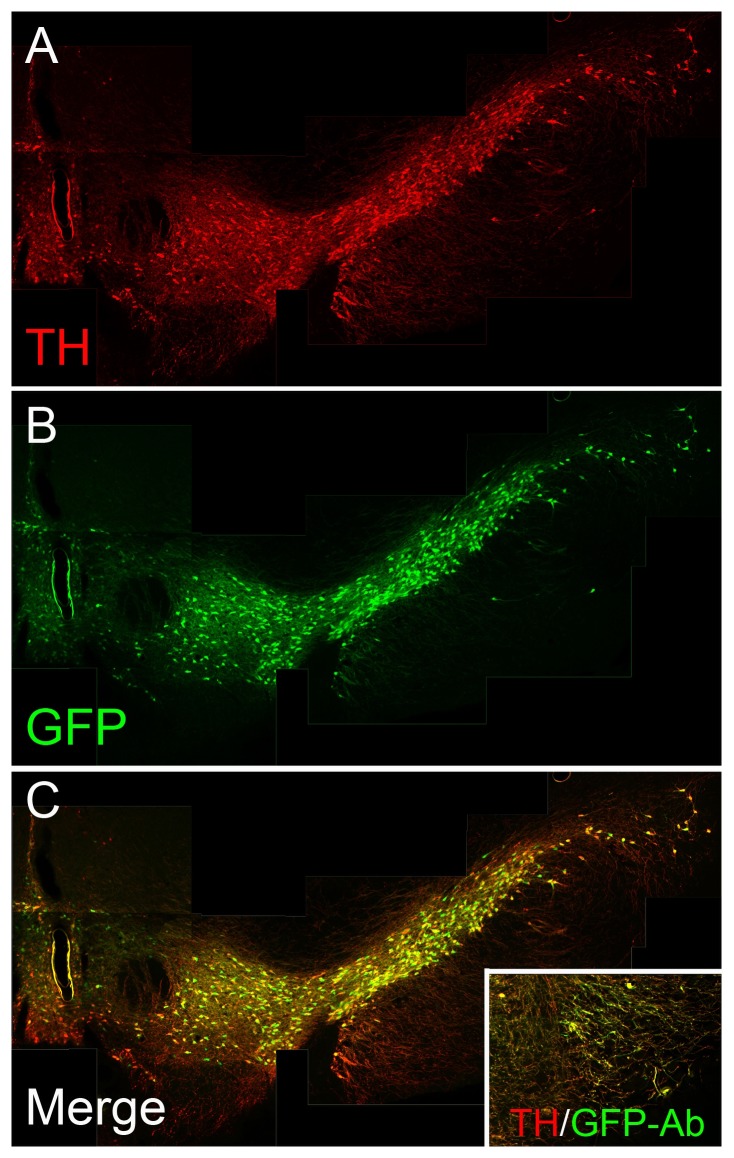
hTH-GFP expression correlates with TH expression in the adult midbrains of Line 12141. TH staining (A) co-labels with GFP (B) expression in midbrain areas, especially in the cell bodies (C) of the SNpc and VTA. The inset of Panel (C) shows that GFP antibody can detect GFP signals in the fine dendritic processes emanating from the cell bodies into the SN pars reticulata (SNpr), which are not as easily visualized by GFP fluorescence in Panel C.

**Figure 2:**
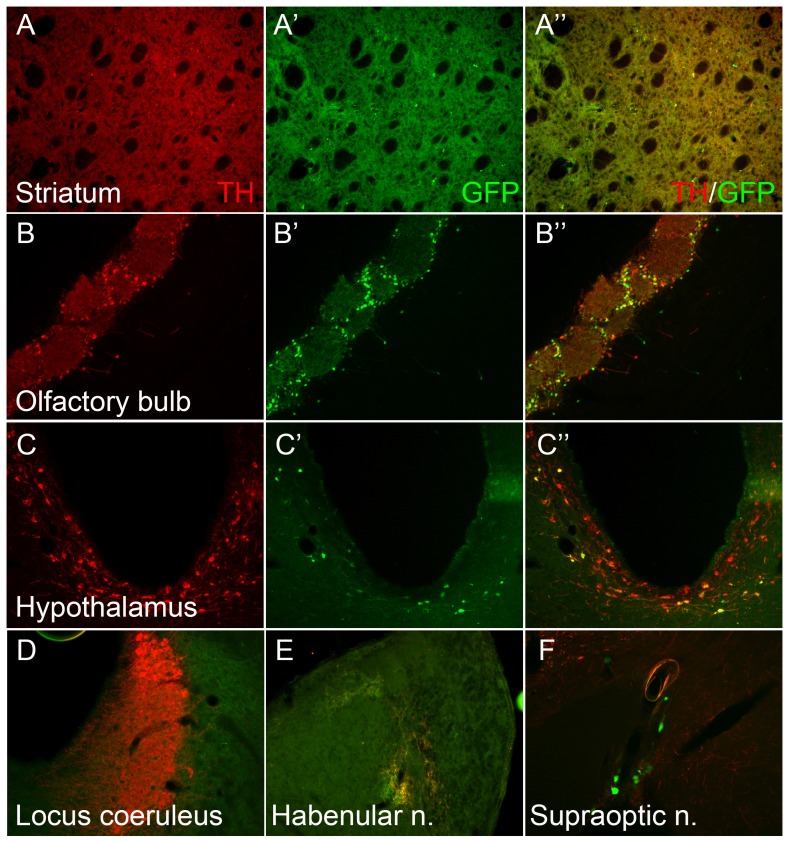
hTH-GFP expression correlates with TH expression in some but not all DA target regions. TH staining co-labels with GFP expression in adult striatum (A-A″) and olfactory bulb (B-B″) in Line 12141. However, GFP expression was less evident in the hypothalamus (C-C″) and absent in the locus coeruleus (D) despite robust TH staining. Ectopic GFP expression is seldom observed in non-TH expressing areas such as habenular nucleus (E) and supraoptic nucleus (F) in Line 12141.

Five founder rat lines were generated using phTH-11 kb-EGFP construct [5]. Brains from both male and female adult individuals from each line were sectioned and immunostained for TH. A summary of results comparing TH staining and GFP expression is provided in Table 1. Since line 12141 showed high faithful expression of GFP in most catecholaminergic (CA) regions and was virtually devoid of ectopic GFP expression in the brain (Fig. 1, 2; Table 1), it was used in the remainder of the analyses below.

**Table 1:** Comparison of TH and GFP expression throughout the CNS and PNS in various hTH-GFP transgenic rat lines.

Rat Line	12108		12121		12141		12142		12155	
	TH staining	GFP	TH staining	GFP	TH staining	GFP	TH staining	GFP	TH staining	GFP
**Traditional CA sites**										
Hypothalamus (A11–14)										
Arcuate n.	+++	-	+++	+	++	-	+++	-	++	-
Periventricular n.	++	+	+	+	++	+	++	-	++	-
Paraventricular n.	++	+	++	+	++	-	++	-	++	+
Zona incerta	+++	+++	+++	+++	+++	+++	+++	-	+++	++
Olfactory bulb	+++	+++	+++	+++	+++	+++	+++	-	+++	+
Substania nigra (A9)	+++	+++	+++	+++	+++	+++	+++	+	+++	+++
VTA(A10)	+++	+++	+++	+++	+++	+++	+++	+	+++	+++
Dorsal raphe n.	+	-	++	+	++	++	++	-	+	-
Locus ceruleus (A6)	++	-	++	-	++	-	++	-	++	-
A5	++	-	++	++	++	+	++	-	++	-
A2	++	+	++	++	++	+	++	-	++	-
A1	++	++	++	++	++	+	++	-	++	-
Area postrema	++	+	++	++	++	-	++	-	++	-
Adrenal gland	ND	ND	ND	ND	++	-	++	-	++	-
										
Striatum	++	++	++	++	++	++	++	-	++	++
										
**Nontraditional CA sites**										
Anterior olfactory n.	-	+	-	+++	-	+	-	-	-	-
N. accumbens	-	+	-	+	-	-	-	-	-	-
Hippocampus	-	+	-	+	-	-	-	-	-	-
Cortex	-	+	-	+	-	-	-	-	-	-
Supraoptic n.	-	+	-	++	-	+	-	-	-	-
Habenular n.	-	++	-	++	-	-	-	-	-	-
Medial mammillary n.	-	+	-	+	-	-	-	-	-	-

Levels of expression are indicated from lowest (-) to highest (+++). ND  =  not determined.

A detailed examination of staining in line 12141 revealed near total overlap (Fig. 1C) in TH (Fig. 1A) and GFP fluorescence (Fig. 1B) in the midbrain DA cell bodies of the SNpc and VTA. Although the fine dendritic processes in the SN pars reticulata (SNpr) were not easily visualized by GFP fluorescence, they were readily detected by staining with GFP antibodies (see Fig. 1C inset). Importantly, the nigrostriatal terminals in the striatum exhibited completely overlapping TH staining and GFP fluorescence (Fig. 2A-A″). With respect to other brain DA nuclei, periglomerular neurons in the olfactory bulb showed a high degree of correlation (Fig. 2B-B′) while hypothalamic DA neurons showed little GFP fluorescence in TH+ cell bodies (Fig. 2C-C″). In contrast with the SNpr, in the hypothalamus and LC, the fluorescence signal was not amplified further by staining with GFP antibodies (data not shown).

Of the four other founder rat lines, three (lines 12108, 12121, 12155) exhibited high level specific GFP fluorescence in DA brain structures, one line (12155) exhibited GFP only in female rats and one line (12142) was totally lacking in GFP expression in the brain. Of the 3 high-expressing lines, robust transgene expression was detected in most CA-rich tissues, including SNpc and VTA (Fig. S1), striatum, olfactory bulb and hypothalamus (Fig. S2).

Importantly, the noradrenergic neurons of the LC (Fig. 2, Fig. S2) and the adrenal medulla (not shown) in all lines were lacking in EGFP fluorescence as also noted in transgenic mouse model generated with the same genetic construct [5], suggesting that the promoter piece is missing key cis elements in the regulation of TH in the LC. As with other transgenic animals using rat [1]–[3] or human [4], [5] TH promoter pieces to drive reporters, ectopic GFP expression was detected in a number of non-CA regions, particularly in lines 12108 and 12121 (Table 1).

### Genetic Characterization of Line 12141

During expansion breeding and characterization of line 12141, animals were mated hTH-GFP female with wildtype SD male, or the reciprocal, wildtype SD female with hTH-GFP carrier male. When wildtype SD females were bred with carrier hTH-GFP males, the percentage of offspring was approximately 50% carrier for the transgene and 50% wildtype, as expected. However, it was noted that hTH-GFP males only generated female offspring that were carriers for the hTH-GFP transgene, while all male offspring were wildtype. Genetic analysis of the inheritance patterns revealed that line 12141 is consistent with insertion of the transgene on the X chromosome (Table S1).

Gene copy number analysis was carried out using qPCR on line 12141 for animals in the N2, N3 and N4 generations of breeding the hTH-GFP carrier with wildtype SD rat at each generation (Fig. S3). The N2 (n = 17 animals) and N3 (n = 12 animals) generations revealed a wide range of copy numbers from 4 to 75 copies detected by qPCR. Carrier animals from the N4 (n = 30 animals) generation showed an average copy number of 23, ranging from 16–33 predicted copies. DNA copy numbers for the hTH-GFP transgene were estimated and normalized relative to the Tert gene as an endogenous control and samples containing 1 copy of GFP and a non-GFP control. The range of estimated copies in the N4 generation was due to fluorescence detected by qPCR which is not absolutely linear with copy number. In the N4 samples there were no dramatic outliers and thus it appears the transgene copy number was stabilizing in the N4 generation while GFP fluorescence in the brain remained strong and specific within midbrain structures as described above.

### Midbrain Development in Line 12141

**Figure 3:**
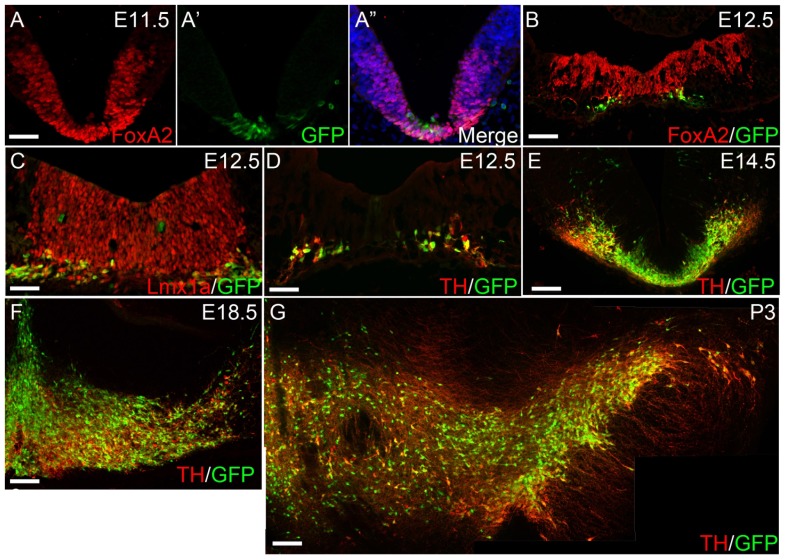
Expression of mDA neuronal markers in hTH-GFP rats at various developmental stages. The mDA progenitor marker Foxa2 (A) is widely distributed along the floor plate of the midbrain at E11.5 while GFP (A′) is detected in only a few midline cells in this region. By E12.5, Foxa2 (B), Lmx1a (C) and TH (D) are all expressed in the central portion of the ventral midbrain. However, Foxa2 expression extends more laterally than Lmx1a. TH expression co-labels with GFP but double-labeled cells are seen only at the base of the midline zone. At all stages examined: E14.5 (E), E18.5 (F) and P3 (G), GFP expression closely approximates TH expression in the midbrain. Scale bar  = 50 µm in A, C, D. Scale bar  = 100 µm in B, E, F, G.

To establish the utility of line 12141 for developmental studies, we examined the location and timing of GFP expression in the midbrain between E10.5-P3 (Fig. 3). We found that GFP+ neurons were first seen at E11.5 when the floor plate marker Foxa2 was clearly evident in the developing ventral mesencephalon (Fig. 3A). By E12.5, overlapping expression of GFP was observed in developing midbrain Lmx1+Foxa2+TH+ DA neurons (Fig. 3B–D) in the central portion of the ventral midbrain. However, Foxa2 expression extended more laterally than Lmx1a and TH expression co-labeled with GFP only at the base of this zone. By E14.5, nearly all GFP+ cells in the midbrain floor plate co-expressed TH (Fig. 3E). With the exception of a small midline area of ectopic GFP (also seen in transgenic mouse models), all SNpc and VTA neurons in the developing midbrain co-expressed GFP and TH at E 18.5 and P3 (Fig. 3F,G).

### Purification of Embryonic Midbrain DA Neurons

**Figure 4:**
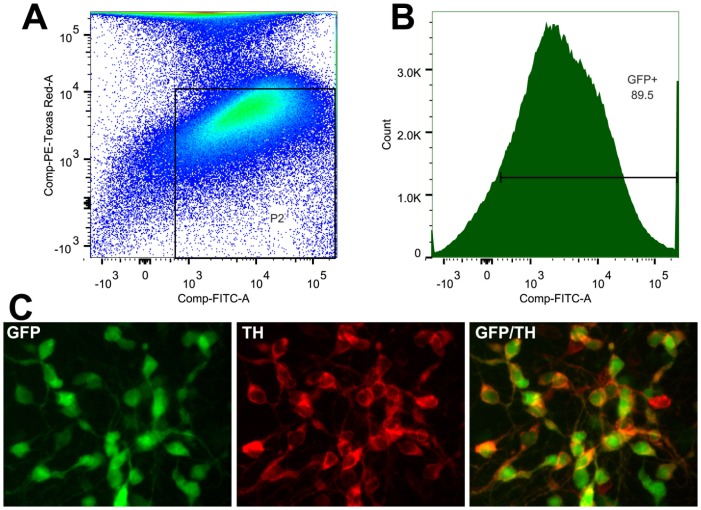
FACS sorting of midbrain DA neurons from E14.5 hTH-GFP rats of Line 12141. (A) Raw data of sorted cells. Gating for live GFP^+^ cells was established by utilizing positive controls for GFP and propidium iodide (PI) stained cells (not shown). The gate, P2, excluded weakly GFP^+^ cells and any PI^+^ (dead) cells. This allowed for the collection of approximately 90% of GFP^+^ cells (B). Collected cells were plated at a density of 300,000cells/well and grown for 3 days, fixed and immunostained for TH (C) exhibit similar co-labeling seen *in vivo* (Figure 1).

To establish that the hTH-GFP rat can be used to purify midbrain DA neurons for studies in culture or for transplants, E14.5 ventral mesencephalic tissue was dissociated and cells were passed through the FACS sorter as described in Methods. Only the pool of healthy PI-negative cells were passed through the cell sorter. As evident in Fig. 4A, GFP fluorescent cells were enriched over 10^4^-fold. FACS analysis revealed the presence of several sub-populations of cells, including a large non-fluorescent cell peak (<1 log GFP), cells with low fluorescence (<10 log GFP) and cells which were highly fluorescent (>10 log GFP). The latter cell population, extended in signal intensity 3–4 logs above control (non-transgenic; data not shown). Thus, the yield of “pure” highly fluorescent cells was approximately 50% of total dissected cells or 89.5% of sorted cells (Fig. 4B). Cells were then collected and grown for 3 days on a complete serum-containing media, as required for purified hTH-GFP mouse DA neurons [17]. When cultures were fixed and immunostained for TH, we found near total overlap in TH+ and GFP+ DA neurons (Fig. 4C). Thus, the hTH-GFP rat can be used to purify midbrain DA neurons (or DA neurons from the SNpc or VTA) for study of PD in culture or for use in transplants.

### Modeling PD in Culture Using the hTH-GFP Reporter Rat

**Figure 5:**
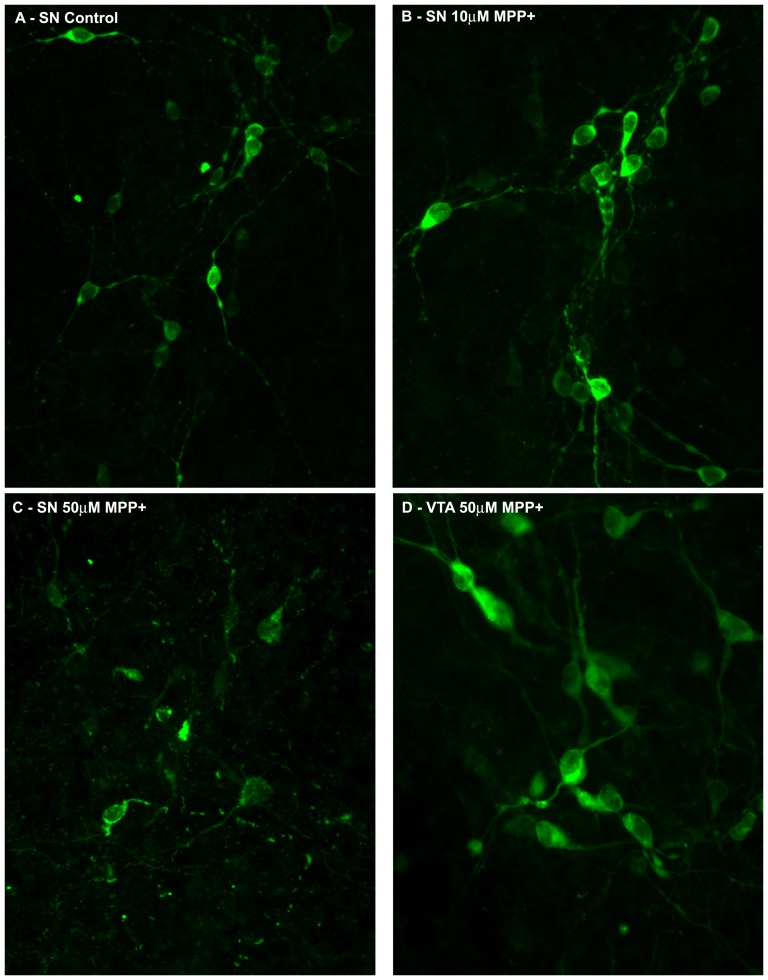
Treatment with MPP^+^ demonstrates selective hTH-GFP^+^ neuron death in the SN *in vitro*. E14.5 hTH-GFP^+^ SN and VTA neurons were plated at a density of 300,000cells/well and grown for 7 days in defined media. (A) Control SN neurons were then treated with media+PBS. (B-C) SN neurons were treated with MPP^+^ at 10 and 50 µM for 48 hours. TH+ neurons treated with 10 µM MPP+ (B) show healthy, undamaged morphology, while 50 µM (C) treated SN neurons are significantly degenerated compared to control (p<0.05). Control (not shown) or 50 µM MPP+ treated (D) VTA neurons exhibit normal, healthy morphology without a significant decline in TH+ cell number.

One of the goals of this work is to utilize transgenic rats to study PD in culture and to develop a rapid high throughout assay to test potential new therapies in vitro. In these studies the SNpc and VTA regions from the E14.5 transgenic rat mesencephalon were microdissected. Region-specific tissues were pooled from multiple embryos, dissociated and plated as described in Methods. Seven days after plating, cultures were administered various concentrations of the DA toxin MPP+ for 48 hours, then fixed and stained. Neurons treated with 10 µM MPP+ (B) show healthy, undamaged morphology, while 50 µM (C) treated SN neurons are fragmented and dead. We found that a dose-dependent response such that 50 µM MPP+ (but not 10 µM MPP+) resulted in the degeneration of nearly all TH+GFP+ DA neurons in SNpc cultures (Fig5A–C) (Control SN: 106 ± 18 TH+ cells/field vs 50 µM MPP+ SN: 45±7 TH+ cells/field; p<0.05). Importantly, DA neurons in VTA cultures remained unaffected even at high doses of MPP+ (Fig. 5D). Thus, as expected, SNpc DA neurons were far more vulnerable to DA specific toxins than VTA DA neurons (Control VTA: 414±21 TH+ cells/field vs 50 µM MPP+ VTA: 423+26 TH+ cells/field; N.S.). Our results indicate that the hTH-GFP rat allows accurate modeling of PD-relevant selective DA neuron death with neurotoxins in culture.

### 6-OHDA-Treated hTH-GFP Reporter Rat Model of PD *In Vivo*

**Figure 6:**
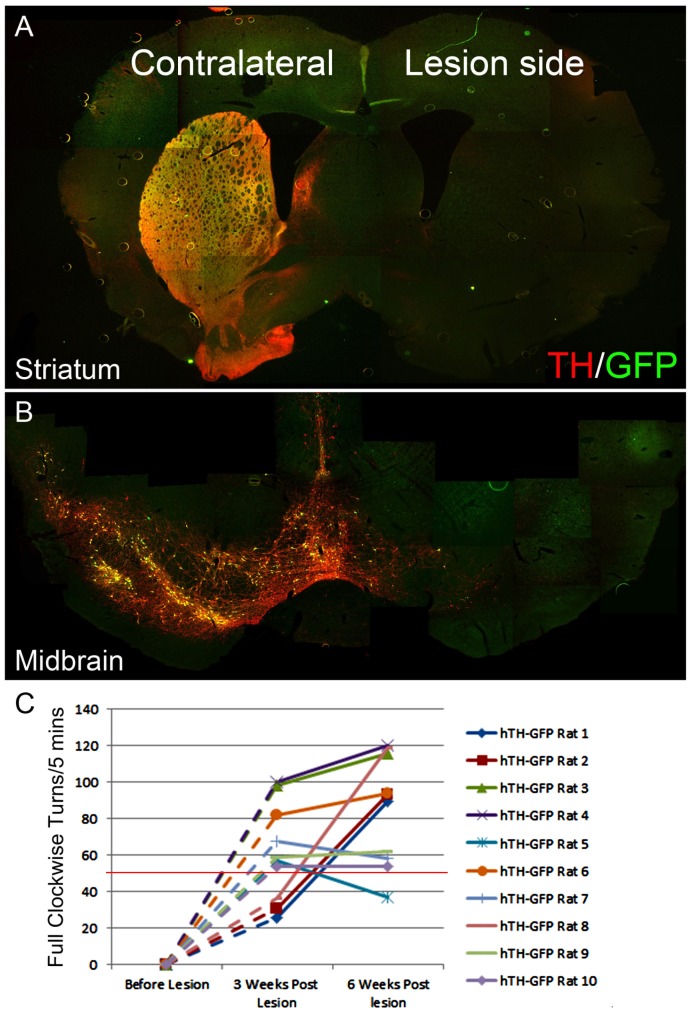
6-OHDA-treated hTH-GFP reporter rat model of PD *in vivo*. Six weeks after 6-OHDA lesion, GFP or TH expression is undetected on the lesion side while both GFP and TH expression is unaffected in the striatum (A) and midbrain (B) on the contralateral side. Rotation tests following 5 mg/kg ip amphetamine challenge were performed at 3 weeks and 6 weeks after lesion. The scores are listed and plotted in Panel (C). In 9 of 10 cases, rats exhibit a robust lesion with over 50 full clockwise turns per 5 minutes. Many rats reached maximum level after 3 weeks (rats 3,4,6,7,9,10), others after 6 weeks (rats 1,2,8). Only one rat (rat 5) showed spontaneous recovery of function after 6 weeks.

As the 6-OHDA rat is the most commonly studied model of PD, we next sought to establish that the hTH-GFP rat could accurately model aspects of the disease in vivo. Rats (N = 10, 5 male and 5 female) were stereotaxically and unilaterally injected with 6-OHDA into the MFB as indicated in Methods (Fig. 6). Rotational motor behavior was assessed after amphetamine challenge prior to 6-OHDA and 3 and 6 weeks after 6-OHDA lesion. Of the 10 rats tested, 9 of 10 were considered to have large (ie. rotational score ≥5 ipsilateral turns/min) lesions at 6 weeks (Fig. 6C). Interestingly, the motor deficit in the 3 rats (rats 3, 4, 6) exhibiting the greatest numbers of ipsilateral turns/min was seen as early as 3 weeks and remained stable between 3 weeks and 6 weeks. Only one rat (rat 5) with a lesion at 3 weeks showed signs of spontaneous recovery at 6 weeks. Rats were then sacrificed and the brains stained. As expected, the observed motor deficits were accompanied by a concomitant loss of nearly all DA cell bodies from the SNpc and terminals in the striatum at 6 weeks on the side ipsilateral to the lesion as compared to the contralateral side (Fig. 6A,B).

### Transplantation of hTH-GFP Fetal DA Neurons in a Rat Model of PD *In Vivo*

**Figure 7:**
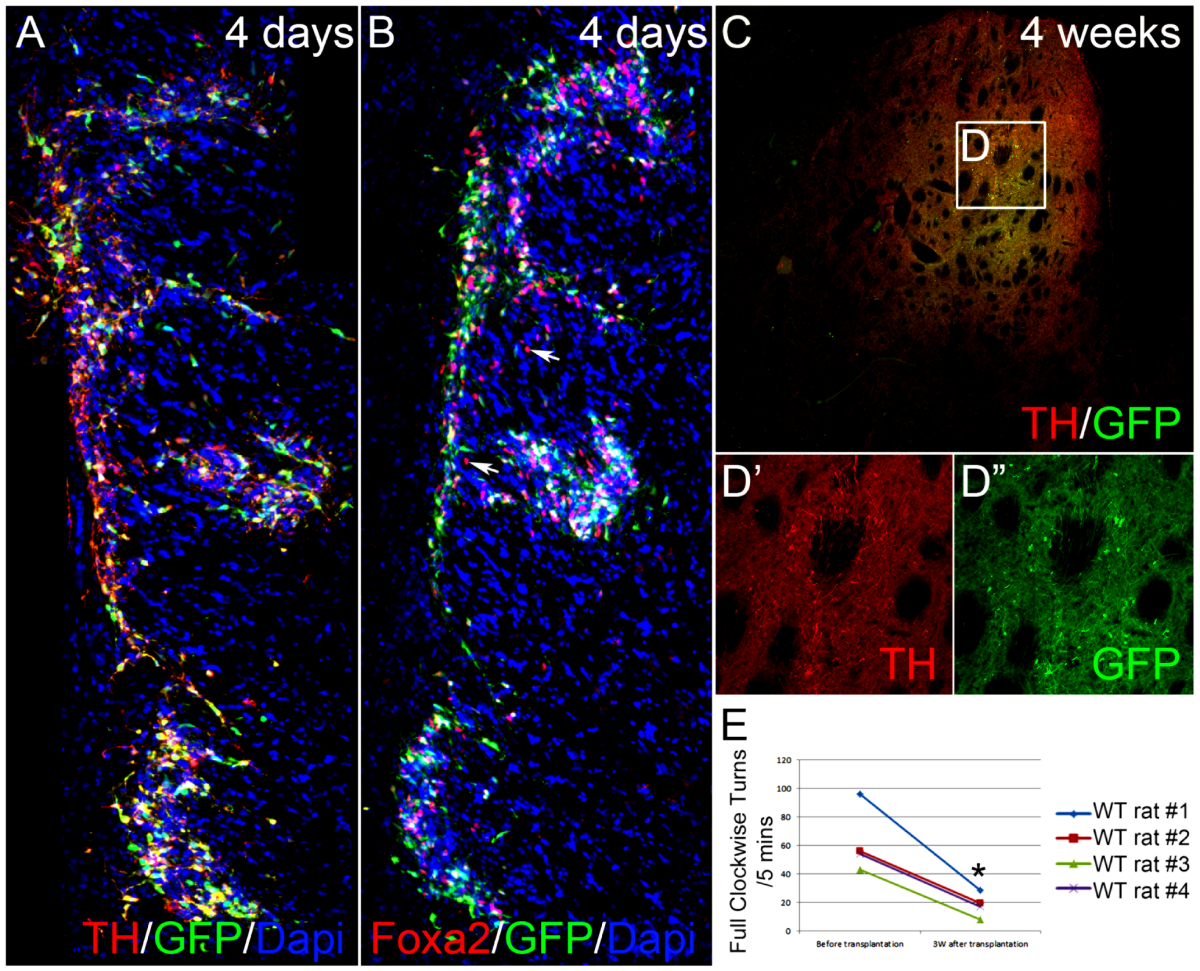
Transplantation of E14.5 hTH-GFP midbrain cells into the striatum of wild type 6-OHDA-lesioned rats. (A–B) At 4 days after transplantation, grafted cells are found along the needle track and at the three cell deposit sites. The majority of transplanted cells are TH+ (A) and Foxa2+ (B). In addition to GFP+ cells, Foxa2+/GFP- dopaminergic progenitor cells are also found in the graft (B). (C) Importantly, TH+/GFP+ processes are seen in the striatum on the ipsilateral lesioned side 4 weeks after transplantation (unlike non-transplanted controls- see Fig. 6A). The magnified view of white box is shown in (D′) and (D″). This section is rostral to the needle track, thus the extensive network of TH+ processes despite the presence of only a few grafted cells. (E) Rotation test scores following 5mg/kg ip amphetamine challenge substantially decreased in all 4 rats 3 weeks after transplantation, consistent with functional recovery from transplantation of fetal hTH-GFP+ mDA neurons; * p<0.05, paired samples t-test.

In this study, cells that had been microdissected from the E14.5 hTH-GFP mesencephalon were transplanted into the striatum of wild type 6-OHDA-lesioned rats. Rats were sacrificed 4 days or 4 weeks later and stained for DA markers Foxa2 and TH. At 4 days after transplantation, grafted cells were observed along the needle track and at the three cell deposit sites. Importantly, the vast majority of transplanted cells were GFP+TH+ (Fig. 7A) or GFP+Foxa2+ (Fig. 7B). In addition, Foxa2+/GFP- DA progenitor cells (which do not yet express TH or GFP) were also found in the graft (Fig. 7B). By 4 weeks after transplantation, TH+/GFP+ processes were widely observed in the ipsilateral striatum even in regions at a distance from the needle track and the majority of transplanted cells (Fig. 7C, D′, D″). Concomitantly, the motor deficit (ie. rotation score) substantially decreased in rats after transplantation (Fig. 7E; 62.4±11.6 before transplantation vs 18.5±4.2 three weeks post transplantation p<0.05), consistent with functional recovery from transplantation of fetal hTH-GFP+ midbrain DA neurons after 3 weeks. The total numbers of surviving TH+ dopamine neurons in grafts were listed in Table 2.

**Table 2:** Rotation scores and Detected TH+ cells in grafts of 4 transplanted rats.

Rat ID	Before transplantation Turn/5 mins	3W after transplantation Turn/5 mins	Detected TH+ cell numbers in one eighth of graft sections (4W)
1	96	28.7	796
2	56.3	19.7	1109
3	43.3	8.3	852
4	54	17.3	669

## Discussion

The results of this study indicate the hTH-GFP reporter rat should serve as an important new model, adding to our arsenal of tools used to study and treat PD. Employing the hTH-GFP reporter construct used previously to create both a transgenic reporter mouse [5] and reporter human embryonic stem cells [18], we demonstrate here that the hTH-GFP rat, particularly line 12141, exhibits high level specific GFP fluorescence in DA brain structures (ie. SNpc, VTA, striatum, olfactory bulb, hypothalamus) with minimal ectopic expression elsewhere in the brain. The pattern of GFP expression driven by the hTH-promoter matches/overlaps the expression pattern of endogenous TH.

These hTH-GFP rats provide several major benefits over their wild type counterparts. First, they allow for the microdissection of the embryonic mesencephalon in a fluorescence microscope, making it possible to segregate PD-susceptible DA neurons of the SNpc from PD-resistant DA neurons of the VTA for studies of disease pathogenesis in culture. The importance of this advantage cannot be over-emphasized as currently midbrain cultures contain a mixture of DA neurons, predominantly comprised of VTA DA neurons which greatly outnumber DA neurons of the SNpc, significantly confounding data analysis. Our studies using the DA-specific toxins MPP+ further indicate that it will be possible to accurately model PD-like neurodegeneration in culture and further dissect the role of environmental modifiers of disease.

A second and related benefit of the hTH-GFP reporter rat is the ability to further purify GFP+ DA neurons by FACS sorting, dramatically enriching their yield in culture from 1-5% seen after dissection of wild type rats [19] versus approximately 90% after microdissection and FACS. By isolating homogeneous rat SNpc or VTA DA neurons, it will be possible to develop high throughput screens to test potential new PD drugs and to automate GFP fluorescence as a quantifiable readout.

Thirdly, our developmental studies indicate that hTH-GFP rat midbrain neurons develop as predicted from murine studies [20]–[26], arising from a population of Foxa2+ floor plate cells and ultimately giving rise to TH+GFP+ DA neurons. Importantly, because GFP expression is more easily detected than TH immunostaining at early developmental stages, the reporter rat may be particularly useful for early embryonic studies. Furthermore, the ability to isolate developing SNpc and/or VTA DA neurons also made possible their study in the brain following transplantation into wild type rats. As further proof that hTH-GFP+ DA neurons develop normally, transplanted GFP+ midbrain cells co-expressed Foxa2 and TH and produced functional motor recovery in the lesioned rat.

Finally, the hTH-GFP reporter rat will facilitate studies on the most widely used in vivo model of PD, the 6-OHDA rat. The large size of the rat versus mouse brain makes stereotaxic procedures (ie. brain administration of toxins like 6-OHDA or MPTP, transplantation) easier. Moreover, in toxin-treated and control rats, the visualization of GFP fluorescent DA neurons will aid researchers using live-cell imaging approaches and for physiological and biochemical (microdialysis) measurements, particularly in slice preparations. Following sacrifice, the isolation of fluorescent brain regions will enable postmortem analyses of distinct DA nuclei (SNpc, SNpr, VTA) and axonal projections (striatum) relevant to the PD brain.

MJFF remains committed to providing the most optimal tools to PD researchers, and we believe this model, which is available from Taconic without restrictions on use to the entire academic and industrial research community, should be valuable for Parkinson's disease research.

## Supporting Information

Figure S1 **hTH-GFP expression (green) matches TH expression (red) in the adult midbrains of Line 12108 (A), Line 12121 (B), females of Line 12155 (D).** However, GFP is poorly expressed in the adult midbrains of Line 12142 (C) and the males of Line 12155(E).
(TIF)

Figure S2 **hTH-GFP expression co-labels TH processes in adult striatum of Line 12108 (A), Line 12121 (D) and females of Line 12155 (J).** However, ectopic GFP expression is observed in the olfactory bulbs of Line 12108 (B), Line 12121 (E) and females of Line 12155 (K). Very few GFP cells are detected in the hypothalamus of all three lines (C, F, L). GFP expression is nearly absent in the striatum (G) and hypothalamus (I) of Line 12142.
(TIF)

Figure S3 **GFP transgene DNA copy number analysis was carried out by qPCR.** X axis shows predicted copy number and y axis is animal ID. Predicted copy number is graphed highest to lowest for each generation examined. N2 (n = 17 animals) generation range is 4–75 copies, N3 (n = 12 animals) generation range is 18–74 copies, N4 (n = 30 animals) generation range is 16–33 copies.
(TIF)

Table S1 **Line 12141 Demonstrates X-Linked Mendelian Inheritance.** Line 12141 NTac:SD-Tg(TH-EGFP)24Xen carries the transgene on the X chromosome, and follows Mendelian inheritance pattern. No autosome insertion has been observed. As shown in the table, male carriers only passed the transgene to daughters, never to sons, while female carriers passed to both gender offspring. Genotype Codes W – Wild type, C – Carrier of the transgene.
(DOCX)
