# Supplementary figures and images for: The hTH-GFP Reporter Rat Model for the Study of Parkinson's Disease
Source: PLoS One. 2014 Dec 2;9(12):e113151. doi: 10.1371/journal.pone.0113151 (PMC4251919; doi:10.1371/journal.pone.0113151)

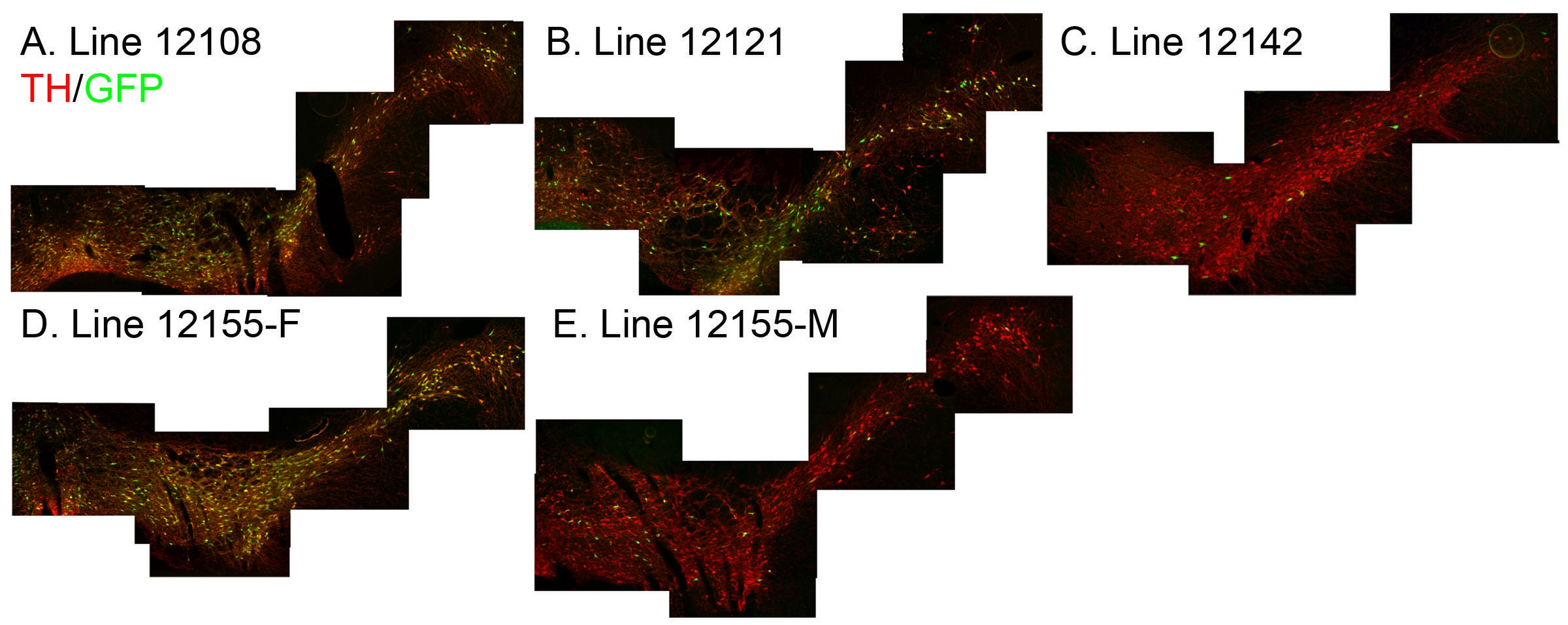

Supplement: Figure S1 — hTH-GFP expression (green) matches TH expression (red) in the adult midbrains of Line 12108 (A), Line 12121 (B), females of Line 12155 (D). However, GFP is poorly expressed in the adult midbrains of Line 12142 (C) and the males of Line 12155(E). (TIF) [file pone.0113151.s001.tif]

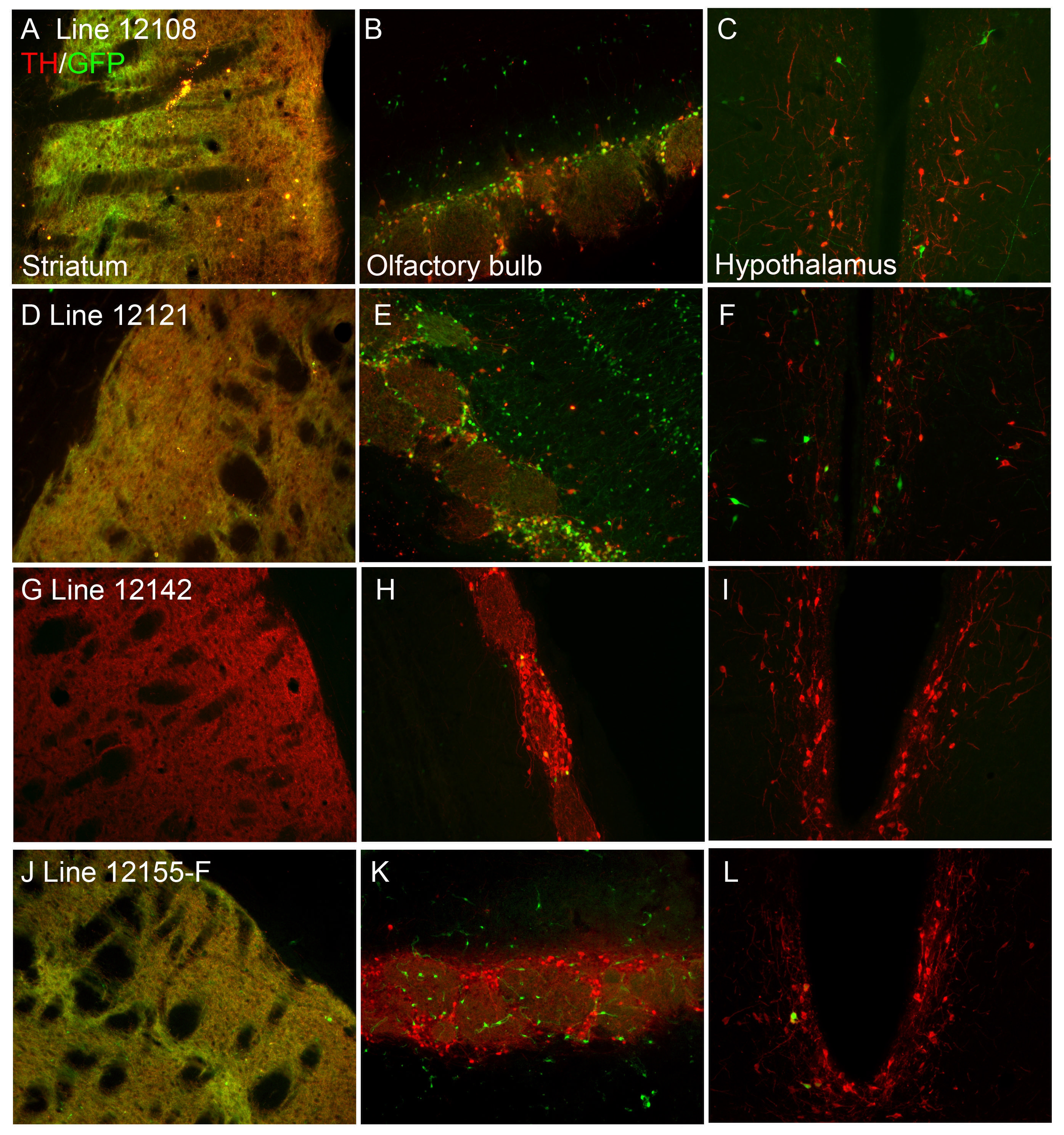

Supplement: Figure S2 — hTH-GFP expression co-labels TH processes in adult striatum of Line 12108 (A), Line 12121 (D) and females of Line 12155 (J). However, ectopic GFP expression is observed in the olfactory bulbs of Line 12108 (B), Line 12121 (E) and females of Line 12155 (K). Very few GFP cells are detected in the hypothalamus of all three lines (C, F, L). GFP expression is nearly absent in the striatum (G) and hypothalamus (I) of Line 12142. (TIF) [file pone.0113151.s002.tif]

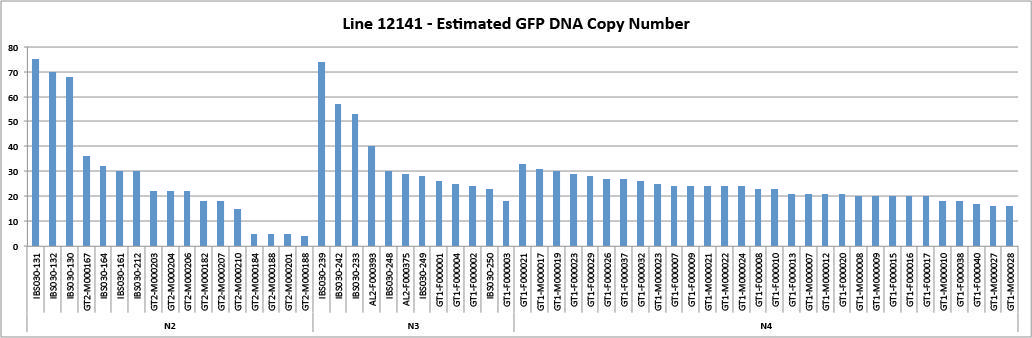

Supplement: Figure S3 — GFP transgene DNA copy number analysis was carried out by qPCR. X axis shows predicted copy number and y axis is animal ID. Predicted copy number is graphed highest to lowest for each generation examined. N2 (n = 17 animals) generation range is 4–75 copies, N3 (n = 12 animals) generation range is 18–74 copies, N4 (n = 30 animals) generation range is 16–33 copies. (TIF) [file pone.0113151.s003.tif]
