# Supplementary material for: The hTH-GFP Reporter Rat Model for the Study of Parkinson's Disease
Source: PLoS One. 2014 Dec 2;9(12):e113151. doi: 10.1371/journal.pone.0113151 (PMC4251919; doi:10.1371/journal.pone.0113151)
Supplement: Table S1 — Line 12141 Demonstrates X-Linked Mendelian Inheritance. Line 12141 NTac:SD-Tg(TH-EGFP)24Xen carries the transgene on the X chromosome, and follows Mendelian inheritance pattern. No autosome insertion has been observed. As shown in the table, male carriers only passed the transgene to daughters, never to sons, while female carriers passed to both gender offspring. Genotype Codes W – Wild type, C – Carrier of the transgene. (DOCX) [file pone.0113151.s004.docx]

**Supplementary Table 1** Line 12141 Demonstrates X-Linked Mendelian Inheritance

| **line 12141** | | | **Female** | | **Male** | |
| --- | --- | --- | --- | --- | --- | --- |
|  |  |  | **C** | **W** | **C** | **W** |
| **mating format (female x male)** | **C x W** | **observed** | **59** | **55** | **57** | **67** |
|  |  | autosomal expected | 57 | 57 | 62 | 62 |
|  |  | X-linked expected | 57 | 57 | 62 | 62 |
|  | **W x C** | **observed** | **32** | **0*** | **0** | **35** |
|  |  | autosomal expected | 16 | 16 | 17.5 | 17.5 |
|  |  | X-linked expected | 32 | 0 | 0 | 35 |
| *2 samples resulted as W, unable to confirm most likely these were mis-sexed not included in this analysis | | | | | |  |

Line 12141 *NTac:SD-Tg(TH-EGFP)24Xen* carries the transgene on the X chromosome, and follows Mendelian inheritance pattern. No autosome insertion has been observed. As shown in the table, male carriers only passed the transgene to daughters, never to sons, while female carriers passed to both gender offspring. Genotype Codes W – Wild type, C – Carrier of the transgene.
